# The Blue Delirium: Serotonin and Anticholinergic Toxicity in a 95‐Year‐Old Woman

**DOI:** 10.1111/jgs.70481

**Published:** 2026-05-02

**Authors:** Camila S. Badell, Kathleen Brodowski, Eloy F. Ruiz, Eric Tien Yen Chyn

**Affiliations:** ^1^ Geriatric Evidence & Research Initiative (GERI‐AMAUTA), Section of Geriatric Medicine, Department of Medicine Rutgers New Jersey Medical School Newark New Jersey USA

**Keywords:** delirium, drug‐related side effects and adverse reactions, polypharmacy, serotonin syndrome

## 1 Patient Story

Mrs. A, a 95‐year‐old woman with essential hypertension, coronary artery disease status post stent placement, heart failure with reduced ejection fraction, spinal stenosis, chronic back pain, chronic dysuria, and history of falls presented with lethargy and weakness following a fall 2 days prior to admission.

At baseline, she lived with her son and a 24‐h home health aide. She was independent with feeding but required assistance for all activities of daily living and transferred via wheelchair. Her medications included aspirin, atorvastatin, lisinopril, carvedilol, and urogesic blue [1, 2, 3], a urinary analgesic with serotonergic and anticholinergic properties (Table 1). Her son reported progressive cognitive and functional decline in recent months, along with new persecutory delusions.

**TABLE 1:** Components of urogesic blue and pharmacologic actions [1, 2, 3].

Component	Mechanism of action	Clinical implication
Methenamine	Hydrolyzed in acidic urine to formaldehyde	Urinary antiseptic. Minimal cognitive effect; may add to medication burden in polypharmacy
Monobasic sodium phosphate	Acidifies urine, promoting methenamine activation	No direct cognitive effect; caution in multimorbid older adults
Methylene blue	Potent reversible inhibitor of MAO‐A [3]	Produces blue‐green urine [1]. May precipitate serotonin toxicity when combined with serotonergic drugs (e.g., SSRIs, SNRIs, MAOIs, tramadol) [1, 2, 3]
Hyoscyamine	Competitive antagonist at muscarinic acetylcholine receptors	Can cause anticholinergic‐related cognitive decline, delirium, and functional impairment in older adults [2]

Abbreviations: MAO‐A, monoamine oxidase A; SNRIs, serotonin‐norepinephrine reuptake inhibitors; SSRIs, selective serotonin reuptake inhibitors.

**FIGURE 1:**
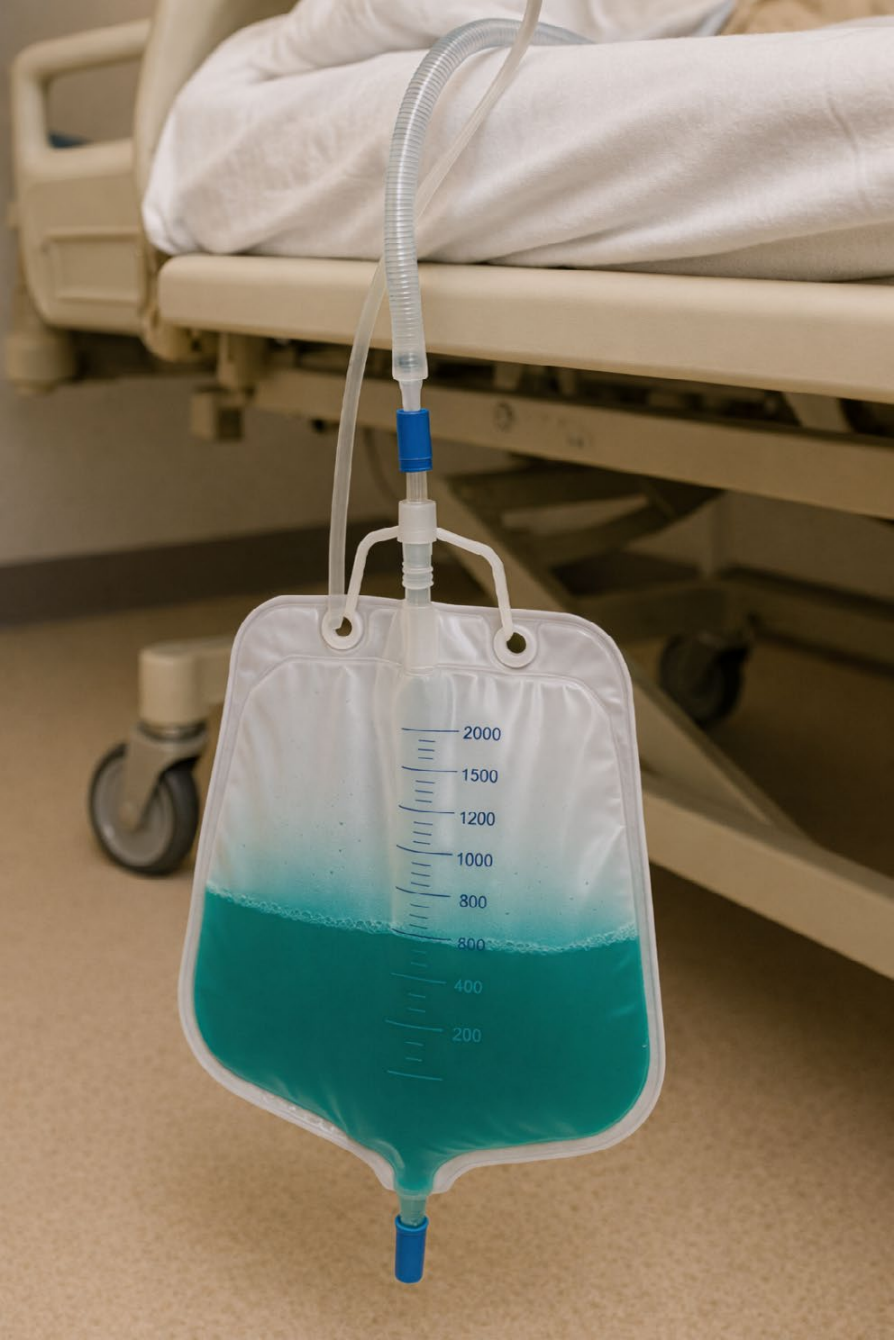
Urine bag with blue‐green colored urine secondary to methylene blue administration. This figure was generated using artificial intelligence (ChatGPT) for illustrative purposes only. It does not depict a real patient, a clinical photograph, or actual patient data.

On admission, she was alert and oriented to self and place, with stable vital signs. Laboratory studies revealed hyponatremia (133 mmol/L), leukocyturia, and nitrite‐positive urine. The urine appeared blue‐green, consistent with her use of urogesic blue (Figure 1). She was started on ceftriaxone for suspected urinary tract infection (UTI). Overnight, she became agitated and inattentive, meeting criteria for delirium. Brain computed tomography (CT) revealed chronic microvascular ischemic changes, volume loss, and stable subdural hygromas consistent with age‐related atrophy.

On hospital Day 2, administration of ondansetron for nausea was followed by the onset of hyperactive delirium with tachycardia, tremors, hyperreflexia, and mild creatine kinase elevation. Repeat CT head showed no acute changes. Given the temporal association and clinical features, serotonin syndrome was suspected due to interaction between methylene blue (a monoamine oxidase inhibitor, MAOI) and ondansetron (a serotonin 5‐HT3 antagonist). Both agents were discontinued, and she was managed with intravenous fluids and supportive care. Her mental status improved over the next 48 h, but intermittent hypoactive and hyperactive delirium persisted. An electroencephalogram was obtained and ruled out seizures. Geriatric Medicine, Neurology and Psychiatry evaluations concluded that her presentation represented delirium superimposed on underlying dementia. Her urine remained blue‐green until hospital Day 4, consistent with methylene blue excretion. Urine culture grew pan‐sensitive *E. coli*, and she completed a 5‐day course of antibiotics.

By discharge, her cognition had improved though mild confusion persisted, consistent with a prolonged delirium recovery. She participated in physical therapy and was discharged to subacute rehabilitation with outpatient geriatric follow‐up.

## 2 Main Teaching Points

This case exemplifies each component of the 5Ms of Geriatric Medicine. Mrs. A's fluctuating mental status highlights the importance of recognizing and managing delirium (*mind*), a common and multifactorial condition often superimposed on preexisting cognitive vulnerability [4, 5, 6, 7]. Her presentation also emphasizes the need for careful medication review (*medications*), as polypharmacy and the interaction between methylene blue and ondansetron led to serotonin syndrome [1, 3, 8], demonstrating how adverse drug effects can contribute to acute confusion in older adults [7, 9]. Her *mobility* limitations, worsened by recent falls and hospitalization, remind us how immobility contributes to functional decline and delays recovery [4]. The case further reflects on *multicomplexity*, where interacting chronic illnesses, infection and medication effects collectively precipitated delirium [4, 7]. Finally, it reinforces the importance of *what matters most*, ensuring that care remains person‐centered and consistent with Mrs. A's goals, values, and quality of life [10].

## 3 Discussion

Delirium is a common and serious Geriatric syndrome, affecting up to 50% of hospitalized older adults and associated with increased mortality, functional decline, and long‐term cognitive impairment [4, 5]. Its pathophysiology is multifactorial, arising from the intersection of predisposing vulnerabilities (such as advanced age and pre‐existing cognitive impairment), and precipitating factors (including infection, metabolic disturbances, medication effects or environmental stressors) [4, 5, 6, 7]. Within the 5Ms framework of Geriatric Medicine, this case highlights the dynamic interplay between mind and medications, while also touching on mobility, multicomplexity and what matters most.

Mrs. A's risk for delirium was amplified by advanced age, chronic microvascular changes, hyponatremia, infection and polypharmacy. Her prolonged cognitive recovery is consistent with evidence that delirium in older adults with baseline cognitive impairment often persists beyond hospital discharge and may result in lasting cognitive deficits [6, 7]. The fluctuating course and incomplete resolution highlight the need to anticipate prolonged recovery trajectories in older adults and to educate families accordingly.

Medication‐related factors played a central role in this case. Polypharmacy and exposure to high‐risk medications remain major contributors to delirium in older adults [7, 9]. Urogesic blue contains methylene blue, a potent MAOI that can precipitate serotonin syndrome when combined with serotonergic agents such as ondansetron [1, 3]. Concurrently, hyoscyamine's anticholinergic properties further increase delirium risk by disrupting cholinergic signaling [1, 2]. Although methylene blue‐induced serotonin syndrome has been commonly reported with parenteral administration, oral formulations remain underrecognized as potential contributors [1].

Recognition of serotonin syndrome in older adults is often challenging because its clinical manifestations often overlap with or mimic delirium itself [8]. In this case, prompt identification and discontinuation of offending agents and supportive care led to gradual clinical improvement, reinforcing the value of early medication review and interprofessional collaboration. Mrs. A's mobility limitations and recent fall further increased her vulnerability, demonstrating how immobility and deconditioning can accelerate both functional and cognitive decline. Her prolonged recovery reflects the inherent complexity of Geriatric syndromes, where multiple medical, functional, and psychosocial factors interact. Ultimately, this case also highlights the importance of focusing on what matters most and aligning care with Mrs. A's goals through shared decision‐making.

## Author Contributions

All authors contributed to the study concept and design, acquisition of data, analysis and interpretation of data, drafting and critical revision of the manuscript. All authors approved the final version of this manuscript.

## Funding

The authors have nothing to report.

## Disclosure

There was no sponsor for this study. No funding source had any role in the design, methods, subject recruitment, data collection, analysis, or preparation of the manuscript.

## Conflicts of Interest

The authors declare no conflicts of interest.
